# Prostate-Specific Antigen Decline Rate in the First Month Is a Timely Predictive Factor for Biochemical Recurrence After Robot-Assisted Radical Prostatectomy

**DOI:** 10.3390/cancers17060961

**Published:** 2025-03-12

**Authors:** Pengfeng Gong, Hisamitsu Ide, Yan Lu, Masayoshi Nagata, Tomoki Kimura, Toshiyuki China, Ippei Hiramatsu, Takuro Kobayashi, Yoshihiro Ikehata, Jun Zhou, Shigeo Horie

**Affiliations:** 1 Department of Urology, Graduate School of Medicine, Juntendo University, Tokyo 113-8431, Japan; gongpengfeng@126.com (P.G.); lyan@juntendo.ac.jp (Y.L.); m-nagata@juntendo.ac.jp (M.N.); to-kimura@juntendo.ac.jp (T.K.); tchina@juntendo.ac.jp (T.C.); ihirama@juntendo.ac.jp (I.H.); ta-kobayashi@juntendo.ac.jp (T.K.); y-ikehata@juntendo.ac.jp (Y.I.); shorie@juntendo.ac.jp (S.H.); 2 Department of Urology, The Third Affiliated Hospital of Soochow University, Juqian Street 185, Changzhou 213004, China; 3 Department of Innovative Longevity, Graduate School of Medicine, Juntendo University, Tokyo 113-8431, Japan; 4 Department of Respiratory & Critical Care, The Third Affiliated Hospital of Soochow University, Juqian Street 185, Changzhou 213004, China; zhouyfan@126.com; 5 Data Science and Informatics for Genetic Disorders, Graduate School of Medicine, Juntendo University, Tokyo 113-8431, Japan

**Keywords:** PSADR1M, BCR, RARP, predictors

## Abstract

**Objectives**: We attempt to assess whether prostate-specific antigen decline rate in the first month (PSADR1M = postoperative PSA in the first month/initial PSA) acts as a predictor for biochemical recurrence (BCR) and to evaluate other preoperative and postoperative variables that may predict BCR following robot-assisted laparoscopic prostatectomy (RARP). **Method**: Based on the D’Amico risk classification system, 777 patients who underwent RARP for localized prostate cancer were classified into a low/intermediate-risk group (*n* = 435) and a high-risk group (*n* = 342). The predictors of BCR were identified by univariate and multivariate logistic regression analyses. The area under the curve (AUC) and optimal cutoff values of PSADR1M were determined by receiver operating characteristic (ROC) analysis. Kaplan–Meier curves for biochemical recurrence-free survival (BRFS) rates were stratified by optimal cutoff values of PSADR1M. **Results**: Effective predictors of BCR in the entire cohort included pT3 (*p* < 0.001), pathological Grade Group (pGG3, pGG4+5) compared to pGG1+2 (*p* < 0.001, *p* = 0.017), positive surgical margins (PSM) (*p* < 0.001), seminal vesicle invasion (SVI) (*p* = 0.006), and PSADR1M ≥ 0.62% (*p* < 0.001). ROC analysis showed that PSADR1M as a predictor for BCR had an AUC of 0.762 for the whole cohort, and 0.821 for the high-risk group, respectively. The optimal cutoff values of PSADR1M were 0.62% in the whole cohort, and 0.68% in high-risk group. **Conclusions**: As an effective predictor of BCR, PSADR1M can assess the tumor status of prostate cancer patients intuitively and effectively after RARP, especially in the high-risk group.

## Simple Summary

In prostate cancer, biochemical recurrence (BCR) refers to a state in which prostate-specific antigen (PSA) levels rise after definitive treatment (such as surgery or radiation therapy), indicating suspected recurrence. BCR differs from clinical recurrence, which can be confirmed through imaging studies; it is an early stage of recurrence primarily detected using PSA levels. At present, no reliable predictors can forecast BCR in prostate cancer patients during the early period after surgery. We have identified a predictive factor as early as the first month after surgery, enabling urologists to provide timely feedback to their patients.

## 1. Introduction

With the increasing use of the robotic system worldwide, robot-assisted laparoscopic prostatectomy (RARP) has become a standard treatment for localized prostate cancer (PCa) in many countries and regions. Although RARP improves urinary control and erectile function compared to open and laparoscopic surgeries, the risk of biochemical recurrence (BCR) following RARP remains significant [1,2]. Previously identified factors predicting BCR include initial prostate-specific antigen (iPSA), positive surgical margins (PSM), gleason scores (GS), pathological tumor stage (pT), body mass index (BMI), family history (FH), age and D’Amico classification, which categorizes patients into low, intermediate, and high-risk groups based on clinical tumor stage (cT), GS, and initial prostate-specific antigen (iPSA) [2,3,4,5,6,7]. 68GaPSMA PRET/CT was introduced as a standard procedure for the diagnosis and staging of high-risk PCa [8]. In a study by Pietro PePe, the GS ≥ 8 rate is 68.8% in the >80-year subgroup, 37.5% in the 76–80-years subgroup, 17.8% in the 71–75-years subgroup, 11.9% in the 61–70-years subgroup, and 3.2% in patients younger than 60 years [6]. As reported in a 2022 study, the BCR rates for GS ≥ 8,GS = 7 and GS = 6 were 75.9%, 20.7%, and 3.4%, respectively, at 3 years of follow-up. In addition, at 3 years of follow-up, 63.4% of high-risk patients, 12.5% of intermediate-risk patients, and 3% of low-risk patients experienced BCR [4]. PSA is used for both screening prostate cancer and evaluating BCR and tumor progression. As reported by prior studies, prostate-specific antigen nadir (PSAn) and the mean time to nadir (TTN) are effective predictors for BCR in patients treated with external beam radiation therapy (EBRT) and androgen deprivation therapy (ADT), cryoablation or radical prostatectomy (RP) [9,10,11,12].

PSA is routinely detected in the first month after RARP, and it can directly and timely reflect the patients’ tumor control status. However, the preoperative iPSA of different prostate cancer patients vary significantly, so using PSA in the first month (PSA1M) to assess the tumor status after RARP is too absolute and simplistic. Therefore, in this study, we detected PSA1M for patients who underwent RARP and calculated the PSA decline rate in the first month (PSADR1M = postoperative PSA in the first month/initial PSA), which is a comprehensive value that compares the preoperative and postoperative tumor burden and can reflect the risk of residual tumors more sensitively. PSADR1M has not been previously studied, and it allowed us to evaluate its potential as an effective predictor for BCR.

## 2. Materials and Methods

### 2.1. Inclusion Criteria

Patients with localized prostate cancer, diagnosed with biopsy pathological and magnetic resonance imaging (MRI) results, are in good health, able to tolerate general anesthesia, and free from severe cardiovascular diseases, other serious complications, and detectable metastases.

### 2.2. Exclusion Criteria

Between July 2013 and December 2023, a total of 1687 localized prostate cancer patients underwent RARP in the department of Urology at Juntendo Hospital, Tokyo. We retrospectively collected patient data and excluded 452 patients who had received neoadjuvant hormone therapy before RARP, 41 patients with follow-up times less than 6 months, 66 patients with non-evaluated perineural invasion, and 351 patients with incomplete data. The remaining 777 patients did not receive adjuvant radiations therapy or hormonal therapy before suffering BCR after RARP.

### 2.3. Risk Group Classification and Study Population

According to D’Amico risk classification, the low-risk group was defined as iPSA ≤ 10 ng/mL, GS ≤ 6, and cT1-cT2a, the intermediate-risk group was defined as 10 ng/mL < iPSA ≤ 20 ng/mL, GS = 7, or cT2b, and the high-risk group was defined as iPSA > 20 ng/mL, GS ≥ 8, or cT2c-cT4 [7].

The 777 patients without neoadjuvant therapy were grouped into two parts based on D’Amico risk classification. The low/intermediate-risk group includes 143 low-risk and 292 intermediate-risk patients, and the high-risk group consists of 342 patients. The preoperative variables include age, iPSA, International Society of Urological Pathology Gleason Grade Group according to biopsy (bGG), clinical tumor stage (cT), and pT, perineural invasion (PNI), International Society of Urological Pathology Gleason Grade Group according to pathological results (pGG), seminal vesicle invasion (SVI), nerve sparing (NS), PSM, PSADR1M, follow-up time, time from RARP to BCR, and BCR rate.

### 2.4. PSA Follow-Up Schedule

PSA was measured by ultrasensitive PSA testing in the 1st and 3rd months after RARP, then every 3 months until 2 years postoperatively, every 6 months in the 3rd year, and annually thereafter. BCR was defined as two consecutive PSA levels greater than 0.2 ng/mL post-surgery. If PSA did not fall below 0.2 ng/mL after RARP, the time of BCR was the date of surgery.

### 2.5. Ethics Statement

This study was conducted in accordance with the Declaration of Helsinki and approved by the Institutional Review Board of Juntendo University Hospital (approval number: E24-0021). All participants provided written informed consent.

### 2.6. Statistical Analysis

Statistical Package for the Social Sciences (SPSS) software, version 29.0 (IBM Corp., Armonk, NY, USA, 2022) was used for statistical analyses. Groups were compared using the Mann–Whitney U test for continuous data, which were presented as a median and IQR, and Pearson’s chi-square test or Fisher’s exact test for categorical data, which were expressed as a percentage. Significant predictors from univariate analysis were further analyzed by multivariate logistic regression. ROC curves for PSADR1M were plotted to calculate the AUC and determine the optimal cutoff values. Kaplan–Meier curves, stratified by optimal cutoff values, were performed to access the BRFS rates. Log-rank tests are used to estimate the relationship between each factor and BRFS rate. All *p*-values were two-sided, with *p* < 0.05 considered statistically significant.

## 3. Results

### 3.1. Preoperative and Postoperative Covariates

Table 1 and Table 2 summarize the preoperative and postoperative covariates for the 777 patients. Preoperative data revealed significant differences between the two groups in Age (*p* = 0.022), iPSA (*p* < 0.001), bGG (*p* < 0.001), and cT (*p* < 0.001). Postoperative data in the high-risk group revealed obvious differences in pT (*p* = 0.001), pGG (*p* < 0.001), SVI (*p* = 0.001), NS (*p* < 0.001), type of LND (*p* < 0.001), follow-up time (*p* < 0.001), BCR rate(*p* < 0.001), and time from RARP to BCR (*p* < 0.001), compared to the low/intermediate-risk group. The median follow-up time and median time from RARP to BCR of the whole cohort was 48 months (IQR 30, 84) and 15 months (IQR 3, 34). Comparing the follow-up times between the two groups, the low/intermediate-risk group had 60 months (IQR 32, 91) versus the high-risk group with 38 months (IQR 24, 72), with a *p* value < 0.001. The time from RARP to BCR in the high-risk group is obviously shorter than that in the low/intermediate-risk group, with 9 months (IQR 0, 30) versus 24 months (IQR 9, 45) (*p* < 0.001). Among the 777 patients, 158 patients experienced BCR, resulting in a BCR rate of 20.3%. The BCR rates between the low/intermediate-risk group and the high-risk group were 15.9% (*n* = 69) versus 26.0% (*n* = 89), with *p* < 0.001, indicating a significant difference.

### 3.2. Univariate Logistic Regression Analysis

As revealed in Table 3, univariate logistic regression analysis identified 9 factors significantly associated with BCR in the entire cohort, including iPSA (*p* = 0.04), cT3 (*p* < 0.001), D’Amico high-risk (*p* = 0.001), pT3 (*p* < 0.001), pGG3 (*p* < 0.001) and pGG4+5 (*p* < 0.017), PSM (*p* < 0.001), SVI (*p* < 0.001), PNI (*p* = 0.003), and PSADR1M (*p* < 0.001). There were 4 effective factors, including pGG3 (*p* = 0.043), pT3 (*p* < 0.001), PSM (*p* = 0.009), SVI (*p* < 0.001), and PSADR1M (*p* < 0.001) identified by univariate logistic regression analysis for predicting BCR in the low/intermediate-risk group (Table 4). In the high-risk group, univariate analysis identified 5 significant variables, including pT3 (*p* < 0.001), pGG3 (*p* < 0.001) and pGG4+5 (*p* < 0.001), PSM (*p* < 0.001), SVI (*p* < 0.001), and PSADR1M (*p* < 0.001) (Table 5).

### 3.3. Multivariate Logistic Regression Analysis

Multivariate logistic regression analysis revealed that pT3 (HR = 2.617, *p* < 0.001), pGG3 vs. pGG1+2 (HR = 2.692, *p* < 0.001), pGG4+5 vs. pGG1+2 (HR = 2.270, *p* = 0.017), PSM (HR = 2.269, *p* < 0.001), SVI (HR = 2.881, *p* = 0.006), and PSADR1M (HR = 2.410, *p* < 0.001) were significant predictors of BCR in the whole cohort (Table 3). As presented by Table 4, pT3 (HR = 2.333, *p* = 0.018), SVI (HR = 6.250, *p* = 0.002), and PSADR1M (HR = 2.584, *p* < 0.001) were effective predictive factors of BCR in the low/intermediate-risk group. In the high-risk group, as revealed by multivariate logistic regression analysis, pT3 (HR = 2.580, *p* = 0.008), pGG3 vs. pGG1+2 (HR = 4.048, *p* < 0.001), pGG4+5 vs. pGG1+2 (HR = 3.737, *p* = 0.002), PSM (HR = 3.120, *p* < 0.001), and PSADR1M (HR = 2.397, *p* < 0.001) were effective predictors of BCR (Table 5).

### 3.4. ROC Curve and AUC for PSADR1M Predicting BCR

**Figure 1:**
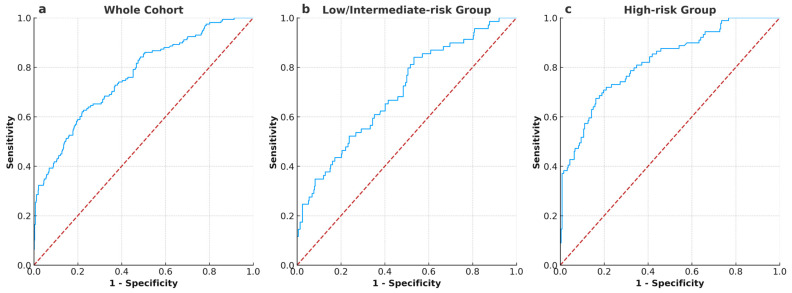
ROC curves and AUC illustrate the performance of PSADR1M in predicting BCR in all groups (**a**) ROC curves and AUC of PSADR1M for the whole cohort, with an AUC of 0.763. (**b**) ROC curves and AUC of PSADR1M for the low- and intermediate-risk group, with an AUC of 0.698. (**c**) ROC curves and AUC of PSADR1M for the high-risk group, with an AUC of 0.821.

ROC analysis showed the AUC for PSADR1M predicting BCR was 0.763 for the whole cohort (Figure 1a), 0.698 for the low/intermediate-risk group (Figure 1b), and 0.821 for the high-risk group (Figure 1c). The optimal cut-off values, which were calculated based on the maximum Youden index, were 0.62% (PSADR1M) for the whole cohort, 0.32% (PSADR1M) for the low/intermediate-risk group, and 0.68% (PSADR1M) for the high-risk group, respectively. According to these optimal cut-off values, the whole cohort, the low/intermediate-risk group, and the high-risk group were classified into two subgroups: PSADR1M < 0.62% and PSADR1M ≥ 0.62%, PSADR1M < 0.32% and PSADR1M ≥ 0.32%, and PSADR1M < 0.68% and PSADR1M ≥ 0.68%, respectively.

### 3.5. Kaplan–Meier Survival Curves for Biochemical Recurrence Free Survival (BRFS) Stratified by PSADR1M

**Figure 2:**
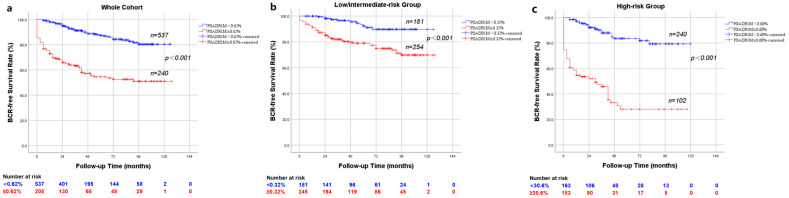
Kaplan–Meier curve for BRFS rates stratified by PSADR1M (**a**) stratified by PSADR1M < 0.62% and PSADR1M ≥ 0.62% in the whole cohort (*p* < 0.001); (**b**) stratified by PSADR1M < 0.32% and PSADR1M ≥ 0.32% in the low/intermediate risk group (*p* < 0.001); (**c**) stratified by PSADR1M < 0.68% and PSADR1M ≥ 0.68% (*p* < 0.001).

The whole cohort (*n* = 777), the low/intermediate-risk group (*n* = 435) and the high/very high-risk group (*n* = 342) were divided into two subgroups: PSADR1M < 0.62% (*n* = 537) and PSADR1M ≥ 0.62% (*n* = 240), PSADR1M < 0.32% (*n* = 181) and PSADR1M ≥ 0.32% (*n* = 254), and PSADR1M < 0.68% (*n* = 240) and PSADR1M ≥ 0.68% (*n* = 102), respectively (Table S1). Table S1 revealed significant differences in BCR rates between each pair of subgroups. In the whole cohort, 158 (20.3%) patients experienced BCR, with 59 (11.0%) and 99 (41.3%) in the PSADR1M < 0.62% subgroup and the PSADR1M ≥ 0.62% subgroup, respectively. In the low/intermediate-risk group, 69 patients (15.9%) experienced BCR, with 11 (6.1%) and 58 (22.8%) in the subgroups, which are grouped based on the optimal threshold value of 0.32%. A total of 89 (26.0%) patients in the high-risk group encountered BCR, with 29 (12.1%) and 60 (58.8%) in each subgroup, respectively. Kaplan–Meier survival curves and log-rank tests showed significant differences between each pair of subgroups with *p* < 0.001(Figure 2a–c).

## 4. Discussion

Our study demonstrated that PSADR1M, defined as the ratio of PSA at the first month post-RARP to the iPSA, serves as a significant and timely predictor of BCR following RARP. We analyzed data from 777 patients who underwent RARP and found that higher PSADR1M values were strongly associated with an increased risk of BCR across the entire cohort, with a particularly pronounced association in the high-risk group. Importantly, we introduced the novel concept of PSADR1M, which has not been previously explored in the context of BCR prediction. Multivariate logistic regression analysis confirmed that PSADR1M is an independent predictive factor, alongside established variables such as pT and SVI. Additionally, ROC analysis showed strong predictive performance of PSADR1M, with optimal cutoff values effectively stratifying patients into different risk categories. These findings highlight the potential of PSADR1M to provide early and sensitive assessment of tumor control, enabling more personalized postoperative management for prostate cancer patients.

Standard therapies for prostate cancer contain radiation therapy [9], cryotherapy [10], surgery [11], hormonal therapy [12], and so on. Dynamics of PSA reduction after ADT and EBRT have obvious differences from those of RP. PSA declines immediately and sharply after RP, reaching its lowest within 4 to 6 weeks, PSA drops gradually after radiation therapy, taking 1 to 3 years to reach its nadir, and PSA decreases quickly after ADT treatment, reaching its nadir level within 3 to 6 months [13]. No matter which therapy is chosen, the risk of BCR is unavoidable. As reported by Kristian D. [14] and Mereya Diaz [15], the 5-year BCR rate and the 10-year BCR rate after RARP can reach up to 20.5% and 26.9%, respectively. Previous researchers have identified several predictors of BCR, such as iPSA, PSAD, PSM, clinical T stage, pathological Gleason score, NCCN risk classification, and so on [16,17,18,19,20].

The post-therapy PSA levels, particularly the nadir PSA (nPSA), and the mean time to PSA nadir (TTN) have been extensively studied. A nPSA ≥ 0.06 ng/mL was identified as a predictive factor for BCR in one study, involving intermediate-risk and high-risk patients treated with EBRT and ADT [9]. The study of Yiyang Liu’s group demonstrated that a nPSA ≥ 0.03 ng/mL and TNN < 3 months are effective predictors for BCR in the high-risk group, following primary whole-gland prostate cryoablation. Chung et al. revealed that a longer TTN was associated with a reduced risk of BCR, and a higher nPSA at 1 or 3 months after Radical prostatectomy (RP) increased the rate of BCR [21]. Additionally, a study from Turkey suggested that both 12-month post-treatment nPSA (nPSA12) > 0.6ng/mL and nPSA > 0.6 ng/mL may independently predict worse BRFS in high-risk prostate cancer patients undergoing EBRT and ADT [22]. In addition to nPSA and TTN, some scholars focus on detectable PSA, which is defined as post-RP PSA ≥ 0.1 ng/mL within 4–8 weeks of surgery [23,24]. A total of 74.4% of patients with detectable PSA after RP will experience PSA progression, increasing the risk of metastasis and death [23]. Compared to patients with an undetectable PSA after surgery, the prognosis of patients with detectable PSA was significantly worse, with a 10-year metastasis-free survival rate of 67% vs. 83% and an overall survival rate of 68% vs. 84% [24]. The previous studies revealed that persistent PSA represented an independent predictor for metastasis and cancer specific death, and salvage radiation (SRT) can improve overall survival (OS) and cancer specific survival (CSS) [25,26]. Although nPSA and TTN are significant predictive factors for BCR, there are some limitations. Firstly, to confirm the PSA nadir usually requires long-term PSA monitoring and comparison with previous and subsequent PSA levels, so that they are unsuitable for timely prediction of BCR. Additionally, if the time when nadir PSA is confirmed is too close to the time of BCR, it may already indicate BCR so that it is unnecessary to predict BCR. This is also why Stephanie L. Skove. and his colleagues excluded the patients with BCR within 6 months after therapy in their study on nPSA and TTN for predicting BCR [11]. Compared with ultrasensitive PSA, detectable PSA, which is defined as post-RP PSA ≥ 0.1 ng/mL within 4–8 weeks of RP, cannot detect subtle changes in PSA at an early stage and fail to provide a timely warning of potential recurrence, missing the upward trend before PSA reaches 0.2 ng/mL and increasing the false-negative rate.

In our study, all 777 patients underwent RARP. Due to its high-resolution 3D visualization, magnified surgical field, excellent precision and stability, favorable working conditions, and high rates of functional recovery, RARP is being increasingly chosen by medical centers and physicians worldwide. Compared to laparoscopic surgery, RARP has shown advantages in controlling urinary incontinence and preserving erectile function, which can be evaluated by patients based on their postoperative symptom [27,28]. However, patients cannot access the tumor control status on their own. Therefore, they often typically inquire doctors about the status of tumor control and outcomes during the first month after RARP. As reported by a previous study, PSA’s half-life is 3.15 days, so it should be undetectable after RP within 21–30 days [29]. PSADR1M (PSA1M/iPSA) is expected to decline to 0.136%, which is calculated based on PSA’s half-life. PSADR1M (PSA1M/iPSA) in this study, calculated according to the maximum Youden index, can more intuitively reflect the extent of the PSA decrease and is a novel predictor that is both timely and effective for predicting BCR and evaluating tumor status. Because the cut-off value of PSADR1M allows for individualized prediction, it is validated by specific clinical data and considers additional clinical factors and individual variations in PSA decline. In addition, BCR requires two consecutive PSA levels greater than 0.2 ng/mL, so that BCR cannot be confirmed until the third month, when PSA has been detected for the second time. This ensures that using PSADR1M to predict BCR does not encounter cases where BCR has already been confirmed.

PSADR1M is a novel predictor for BCR that has not been reported in prior studies. And it is a strong predictive factor of BCR in multivariate logistic regression models, regardless of whether it is applied in the whole cohort, the low/intermediate-risk group, or the high-risk group. In our study, the optimal cutoff value for PSADR1M in the whole cohort was 0.62%. This means that if PSA1M declines by more than 99.38% compared to iPSA, the risk of BCR is significantly lower compared to those whose PSA1M declines less than 99.38%. Similarly, as for the low/intermediate-risk group and the high-risk group, a PSA1M decline of more than 99.68% and 99.32%, respectively, indicates a substantially reduced risk of BCR.

The predictive factors for BCR in previous studies include pT3, pGG ≥ 3, PSM, iPSA > 20 ng/mL, lymph node involvement (LNI), PNI, and so on [30,31]. In our study, only 452 (58.2%) patients underwent LND, and the lymph node positive rate was very low, which could not predict BCR adequately. It is controversial that LNI is an effective predictor of BCR because it is influenced by a lot of factors. Firstly, compared to laparoscopic and open surgeries, more lymph nodes are dissected, and more positive lymph nodes are detected in RARP [32,33]. Secondly, considering the complications associated with lymph node dissection, different urologists may choose different methods (non LND, sLND or extended Lymph Node Dissection, eLND) [34]. Thirdly, patients with typical LNI should receive ADT plus radiation therapy after surgery, which may interfere with BCR [35]. In research from Thailand [30], iPSA > 20 ng/mL is an effective predictor for BCR in multivariate logistic regression analysis, with a *p* value < 0.001. While, both iPSA in our study and iPSA > 20 ng/mL in a French study (*p* > 0.05) [36] were a predictive factor for BCR in univariate logistic regression analysis, neither remained significant in multivariate logistic regression analysis, indicating that iPSA > 20 ng/mL may not as strong an influence as other factors for BCR such as pT3 and PSM. This phenomenon may be caused by short-term follow-up in some patients and the study population varies. Short-term follow-up may not fully evaluate the long-term predictive value of iPSA for BCR [37]. Additionally, variation in the proportion of high-risk patients or low-risk patients across studies may lead to different influence in the predictive value of iPSA for BCR. A very low percentage (2.1%, 16/777) of patients with iPSA > 20ng/mL in the entire cohort may reduce the statistical power of predicting BCR and lead to variability in results.

Our study excluded patients who received neoadjuvant hormonal therapy before RARP, so that potential interference with PSADR1M could be eliminated. We also used ultrasensitive PSA testing, which helps to detect subtle changes in PSA earlier, monitor prostate cancer more accurately and reduce the false-negative rate. Despite these efforts to minimize errors, some are unavoidable. It is a retrospective study, which has inherent limitations, including potential selection and information bias. In this study, patients were selected from a single database, and only those who underwent RARP were included, which may limit the generalizability of our findings. In our retrospective study, some patients were excluded due to incomplete information, which may lead to information bias and affect the final results. The short follow-up time of some patients may not fully capture long-term BCR trends, potentially underestimating BCR rates, because some patients may develop BCR beyond the follow-up period in our study. Furthermore, PSA1M was not strictly standardized, as it could not be precisely measured on the 30th postoperative day for all patients, and some underwent testing on 28 days (4 weeks) or 35 days (5 weeks) so that some measurement variability occurred. These factors should be considered when interpreting the collection and extended follow-up periods are needed to validate our findings.

## 5. Conclusions

Although BCR does not indicate that patients with prostate cancer will immediately progress to death, timely identification of BCR is crucial for slowing disease progression. The early detection of PSA allows for more personalized treatment strategies, improving long-term outcomes. Timely adjuvant hormone therapy and radiotherapy are often obviously effective. In the study, we identified PSADR1M as an effective and timely predictor for BCR across the whole cohort, as well as in the low/intermediate-risk group and the high-risk group. Predicting BCR at an early stage provides urologists with valuable information for risk stratification, enabling proactive treatment planning. From a healthcare perspective, early identification of high-risk patients through PSADR1M may also decrease unnecessary surveillance and overtreatment in lower-risk individuals, potentially reducing healthcare costs. Doctors can use PSADR1M to predict BCR and inform patients during the first month after surgery, developing a more specific follow-up strategy based on individual risk level.

## Figures and Tables

**Table 1:** Statistics for the whole cohort and comparison of preoperative variables between the low/intermediate-risk group and the high-risk group.

Preoperative Covariates	Whole Cohort (*n* = 777)	Low/Intermediate-Risk (143/292) (*n* = 435)	High-Risk (*n* = 342)	*p-*Value
Age (years, median, IQR)	68.0 (63.0, 72.0)	67 (62.0, 71.0)	68.0 (63.0, 73.0)	0.022
iPSA (ng/mL, median, IQR)	6.6 (5.1, 9.0)	6.1 (5.0, 8.3)	7.0 (5.3, 10.0)	0.001
bGG (*n*, %)				0.001
1 (3 + 3 = 6)	240 (30.9)	186 (42.7)	54 (15.8)	
2 (3 + 4 = 7)	237 (30.5)	149 (34.3)	88 (25.7)	
3 (4 + 3 = 7)	160 (20.6)	100 (23.0)	60 (17.5)	
4 (5 + 3, 4 + 4, 3 + 5 = 8)	120 (15.4)	0	120 (35.1)	
5 (5 + 4, 4 + 5 = 9, 5 + 5 = 10)	20 (2.6)	0	20 (5.9)	
Clinical T stage				0.001
T1c	141 (18.2)	130 (30.0)	11 (3.2)	
T2a	287 (36.9)	238 (54.6)	49 (14.3)	
T2b	84 (10.8)	67 (15.4)	17 (5.0)	
T2c	229 (29.5)	0	229 (67.0)	
T3a	31 (4.0)	0	31 (9.1)	
T3b	5 (0.6)	0	5 (1.4)	

iPSA, initial prostate-specific antigen; bGG, biopsy International Society of Urological Pathology grade group.

**Table 2:** Statistics for the whole cohort and comparison of postoperative variables between the low/intermediate-risk group and the high-risk group.

Postoperative Covariates	Whole Cohort (*n* = 777)	Low/Intermediate-Risk (143/292) (*n* = 435)	High-Risk (*n* = 342)	*p-*Value
pT stage (number, %)				
pT1	2 (0.3)	1 (0.2)	1 (0.3)	0.001
pT2a	71 (9.1)	42 (9.7)	29 (8.5)	
pT2b	16 (2.1)	11 (2.5)	5 (1.5)	
pT2c	481 (61.9)	290 (66.7)	191 (55.8)	
pT3a	149 (19.2)	71 (16.3)	79 (23.1)	
pT3b	58 (7.4)	20 (4.6)	37 (10.8)	
PNI (number, %)	352 (45.3)	184 (42.3)	168 (49.1)	0.058
pGG (number, %)				<0.001
GG 1	145 (18.7)	105 (24.1)	40 (11.7)	
GG 2	353 (45.4)	213 (49.0)	140 (40.9)	
GG 3	191 (24.6)	100 (23.0)	91 (26.6)	
GG 4	61 (7.8)	12 (2.8)	49 (14.3)	
GG 5	27 (3.5)	5 (1.1)	22 (6.4)	
PSM (number, %)	223 (28.7)	120 (27.6)	103 (30.1)	0.439
SVI (number, %)	58 (7.5)	20 (4.6)	38 (11.1)	0.001
NS (number, %)				<0.001
NNS	385 (49.6)	162 (37.2)	223 (65.2)	
UNS	193 (24.8)	123 (28.3)	70 (20.5)	
BNS	199 (25.6)	150 (34.5)	49 (14.3)	
Type of LND (number, %)				<0.001
Non LND	315 (40.5)	251 (57.7)	84 (24.6)	
sLND	459 (59.1)	184 (42.3)	255 (74.6)	
eLND	3 (0.3)	0	3 (0.8)	
PSADR1M (%, median, IQR)	0.38 (0.20,0.72)	0.38 (0.21, 0.70)	0.38 (0.19, 0.77)	0.916
Follow-up time (month, median, IQR)	48.0 (30.0,84.0)	60.0 (32.0, 91.0)	38.0 (24.0, 72.0)	<0.001
BCR rate (number, %)	158 (20.3)	69 (15.9)	89 (26.0)	<0.001
Time from RARP to BCR (month, median, IQR)	15 (3, 34)	24 (9, 45)	9 (0, 30)	<0.001

PNI, perineural invasion; pGG, pathological International Society of Urological Pathology Grade Group; PSM, positive surgical margin; SVI, seminal vesicle invasion; NS, nerve sparing; NNS, non-nerve sparing; UNS, unilateral nerve sparing; BNS, bilateral nerve sparing; LND, lymphadenectomy; sLND, standard lymphadenectomy; eLND, extended lymphadenectomy; PSADR1M (PSA1M/iPSA), prostate-specific antigen decline rate in the 1st month (PSA1M/iPSA, postoperative PSA in the 1st month/initial PSA); BCR, biochemical recurrence.

**Table 3:** Univariate and multivariate analysis for predicting biochemical recurrence (BCR) in the whole cohort.

Covariates	Uni-Variate Analysis			Multi-Variate Analysis		
	HR	95% CI	*p* Value	HR	95% CI	*p-*Value
Age	1.008	(0.982, 1.035)	0.548			
iPSA	1.034	(1.001, 1.067)	0.040	0.993	(0.957, 1.030)	0.709
cT1+cT2	Ref			Ref		
cT3	4.553	(2.288, 9.058)	<0.001	1.784	(0.703, 4.529)	0.223
D’Amico Low/intermediate-risk	Ref			Ref		
D’Amico High-risk	1.866	(1.311, 2.656)	0.001	1.062	(0.667, 1.690)	0.800
pT1+pT2	Ref			Ref		
pT3	4.916	(3.393, 7.123)	<0.001	2.617	(1.566, 4.373)	<0.001
pGG 1+2	Ref			Ref		
pGG 3	2.647	(1.763, 3.974)	<0.001	2.692	(1.664, 4.355)	<0.001
pGG 4+5	4.749	(2.891, 7.800)	<0.001	2.270	(1.159, 4.445)	0.017
NSM	Ref			Ref		
PSM	2.595	(1.805, 3.730)	<0.001	2.269	(1.450, 3.549)	<0.001
Non-SVI	Ref			Ref		
SVI	10.349	(5.708, 18.532)	<0.001	2.881	(1.354, 6.131)	0.006
Non-PNI	Ref			Ref		
PNI	1.169	(1.189, 2.404)	0.003	0.866	(0.549, 1.368)	0.538
PSADR1M, %	1.274	(1.133, 1.415)	<0.001	2.410	(1.847, 3.145)	<0.001
PSADR1M < 0.62%	Ref					
PSADR1M ≥ 0.62%	5.688	(3.917, 8.261)	<0.001			

HR, hazard radio; CI, confidence interval.

**Table 4:** Univariate and multivariate analysis for predicting biochemical recurrence (BCR) in the low- and intermediate-risk group.

Covariates	Uni-Variate Analysis			Multi-Variate Analysis		
	HR	95% CI	*p-*Value	HR	95% CI	*p-*Value
pGG1+2	Ref					
pGG3	1.804	(1.018, 3.196)	0.043	1.939	(1.009, 3.724)	0.047
pGG4+5	1.968	(0.613, 6.314)	0.255	1.456	(0.372, 5.706)	0.590
pT1+pT2	Ref					
pT3	3.555	(2.050, 6.165)	<0.001	2.333	(1.153, 4.722)	0.018
NSM	Ref					
PSM	2.034	(1.191, 3.474)	0.009	1.769	(0.954, 3.281)	0.070
Non-SVI	Ref					
SVI	11.906	(4.554, 31.126)	<0.001	6.250	(1.959,19.939)	0.002
PSADR1M (%)	2.607	(1.813, 3.749)	<0.001	2.584	(1.768, 3.777)	<0.001
PSADR1M < 0.32%	Ref					
PSADR1M ≥ 0.32%	4.573	(2.325, 8.996)	<0.001			

NSM, negative surgical margin.

**Table 5:** Univariate and multivariate analysis for predicting biochemical recurrence (BCR) in the high-risk group.

Covariates	Uni-Variate Analysis			Multi-Variate Analysis		
	HR	95% CI	*p-*Value	HR	95% CI	*p-*Value
pT1+pT2	Ref					
pT3	5.857	(3.476, 9.870)	<0.001	2.580	(1.276, 5.219)	0.008
pGG1+2	Ref					
pGG 3	3.884	(2.107, 7.160)	<0.001	4.048	(1.929, 8.495)	<0.001
pGG 4+5	5.928	(3.127, 11.237)	<0.001	3.737	(1.636, 8.540)	0.002
NSM	Ref					
PSM	3.215	(1.935, 5.341)	<0.001	3.120	(1.610, 6.048)	<0.001
Non-SVI	Ref					
SVI	8.288	(3.962, 17.339)	<0.001	1.619	(0.618, 4.245)	0.327
PSADR1M(%)	2.645	(1.854, 3.773)	<0.001	2.397	(1.655, 3.471)	<0.001
PSADR1M < 0.68%	Ref					
PSADR1M ≥ 0.68%	10.394	(5.977, 18.075)	<0.001			

## Data Availability

All data analyzed in this study can be provided by applying to S. Horie and the corresponding author, Hisamitsu Ide.

## Supplementary Materials

The following supporting information can be downloaded at: https://www.mdpi.com/article/10.3390/cancers17060961/s1, Table S1: BCR rates in the whole cohort, the low/intermediate-risk group, and the high-risk group based on the PSADR1M.
