# Supplementary material for: Prostate-Specific Antigen Decline Rate in the First Month Is a Timely Predictive Factor for Biochemical Recurrence After Robot-Assisted Radical Prostatectomy
Source: Cancers (Basel). 2025 Mar 12;17(6):961. doi: 10.3390/cancers17060961 (PMC11940538; doi:10.3390/cancers17060961)
Supplement: Supplementary file 1 [file cancers-17-00961-s001.zip › cancers-3480760-supplementary.pdf]

Table S1. BCR rates in the whole cohort, the low/intermediate-risk group, and the high-risk group based on the PSADR1M.

Group	Whole cohort (n = 777)			Low/Intermediate-risk (n = 435)			High-risk (n = 342)		
PSADR1M (%)	< 0.62 (n = 537)	≥ 0.62 (n = 240)	*P*-Value	< 0.32 (n = 181)	≥ 0.32 (n = 254)	*P*-Value	< 0.68 (n = 240)	≥ 0.68 (n = 102)	*P*-Value
BCR (n, %)	59 (11.0%)	99 (41.3%)	< 0.001	11 (6.1%)	58 (22.8%)	< 0.001	29 (12.1%)	60 (58.8%)	< 0.001
